# Clinical relevance of cell-free mitochondrial DNA during the early postoperative period in kidney transplant recipients

**DOI:** 10.1038/s41598-019-54694-x

**Published:** 2019-12-09

**Authors:** Kipyo Kim, Haena Moon, Yu Ho Lee, Jung-Woo Seo, Yang Gyun Kim, Ju-Young Moon, Jin Sug Kim, Kyung-Hwan Jeong, Tae Won Lee, Chun-Gyoo Ihm, Sang-Ho Lee

**Affiliations:** 1 Division of Nephrology, Department of Internal Medicine, Kyung Hee University, Seoul, Korea; 2 Department of Internal Medicine, Seoul National University Bundang Hospital, Seongnam, Korea; 3 Division of Nephrology, Department of Internal Medicine, CHA Bundang Medical Center, CHA University, Seongnam, Korea; 4 Kyung Hee Medical Science Research Institute, Kyung Hee University, Seoul, Korea

**Keywords:** Acute kidney injury, Prognostic markers

## Abstract

Recent studies indicate that urinary mitochondrial DNA (mtDNA) is predictive of ischemic AKI and is related to delayed graft function (DGF) in renal transplantation. Nevertheless, the clinical implications and prognostic value of urinary mtDNA in kidney transplantation remain undetermined. Here, we aimed to evaluate the associations between cell-free mtDNA and clinical parameters, including pathological findings in allograft biopsy and post-transplant renal function. A total of 85 renal transplant recipients were enrolled, and blood and urine samples were collected at a median of 17 days after transplantation. Cell-free nuclear and mtDNA levels were measured by quantitative polymerase chain reaction for *LPL* and *ND1* genes. Urinary cell-free mtDNA levels were significantly higher in patients with DGF (*P* < 0.001) and cases of deceased donor transplantation (*P* < 0.001). The subjects with acute rejection showed higher urinary mtDNA levels than those without abnormalities (*P* = 0.043). In addition, allograft functions at 9- and 12-month post-transplantation were significantly different between tertile groups of mtDNA independent of the presence of DGF or acute rejection, showing significantly better graft outcome in the lowest tertile group. Urinary cell-free mtDNA levels during the early post-transplant period are significantly associated with DGF, acute rejection in graft biopsy, and short-term post-transplant renal function.

## Introduction

Over the past decades, kidney transplantation has become the treatment of choice for end-stage renal disease, due to improvements in graft outcome. However, there are still significant obstacles preventing further increase in graft survival, such as delayed graft function (DGF) and allograft rejection, which is a major challenge for clinicians. In particular, during the early post-transplant period, the allograft is adversely affected by profound ischemia-reperfusion injury (IRI), which is inevitable but critically affects subsequent graft outcome. IRI is a major risk factor for DGF and is associated with chronic allograft dysfunction and acute rejection (AR)^1–4^. The pathophysiology of IRI includes excessive generation of reactive oxygen species (ROS) and inflammatory responses, resulting in tissue damage and cell death^5^. During these processes, innate immunity is triggered by the endogenous damage-associated molecular patterns (DAMPs)^6^, and the activation of toll-like receptor (TLR) and related signaling pathways has been reported in previous studies^6–8^.

In recent years, mitochondrial DAMPs have received considerable attention as an important mediator of tissue injury in various inflammatory conditions, including trauma, sepsis, cancer, hemodialysis, and transplantation^9–14^. Of these mitochondrial DAMPs, cell-free mitochondrial DNA (mtDNA) has been reported to be a predictive biomarker of the progression of acute kidney injury (AKI)^15,16^. Transplantation studies have demonstrated that increased extracellular mtDNA is associated with elevated levels of inflammatory cytokines, early organ dysfunction in liver transplantation^17^, and DGF in kidney transplantation^18^. These findings suggest that mtDNA could be one of the important DAMPs in organ transplantation. Nevertheless, to date, there is limited research investigating the clinical implications of cell-free mtDNA in kidney transplantation. Moreover, it is uncertain whether the detected mtDNA is merely a consequence of mitochondrial damage from IRI or a causative factor for subsequent graft dysfunction, impacting as DAMP. In the present study, we evaluated the clinical implication of cell-free mtDNA during the early post-transplant period on histological and clinical parameters and examined the association between cell-free mtDNA levels and short-term graft outcome in kidney transplantation.

## Results

### Baseline characteristics of patients

Baseline characteristics of the included patients are presented in Table 1. The mean age of the included patients was 47.4 ± 10.7 years, 57.6% were men, and 76.5% were transplanted from deceased donors. Allograft biopsy was performed with sample collection at around 17 days after transplantation. The median eGFR at baseline was 65.3 mL/min/1.73 m^2^, and tertile 3 group had a lower eGFR than tertile 1 and 2 groups. Ten patients experienced DGF and those in higher tertiles were more likely to experience DGF. Corticosteroids and tacrolimus were administered as initial immunosuppressive therapy in all subjects. There were no significant differences in sex, age, donor category, and dialysis duration between the groups.

**Table 1:** Baseline characteristic of study population according to the tertiles of urinary mtDNA level.

	Total (n = 85)	Tertile 1 (n = 28)	Tertile 2 (n = 28)	Tertile 3 (n = 29)	*P*
**Donor**					
Age, years	47.4 ± 10.7	44.2 ± 10.5	48.6 ± 9.6	49.2 ± 11.7	0.164
Male sex	49 (57.6%)	15 (53.6%)	15 (53.6%)	19 (65.5%)	0.572
Donor type					0.002
Living	20 (23.5%)	13 (46.4%)	4 (14.3%)	3 (10.3%)	
Deceased	65 (76.5%)	15 (53.6%)	24 (85.7%)	26 (89.7%)	
**Recipient**					
Age, yr	50.0 (41.0–56.0)	49.5 (40.0–55.0)	49.0 (41.0–54.5)	51.0 (47.0–60.0)	0.314
Male sex	59 (69.4%)	18 (64.3%)	22 (78.6%)	19 (65.5%)	0.436
BMI, kg/m2	21.9 (20.1–24.2)	22.2 (20.5–24.3)	21.5 (19.8–23.9)	21.9 (19.8–25.1)	0.518
Cause of ESRD, %					0.252
Diabetes	18 (21.2%)	4 (14.3%)	6 (21.4%)	8 (27.6%)	
Hypertension	23 (27.1%)	11 (39.3%)	5 (17.9%)	7 (24.1%)	
Glomerulonephritis	25 (29.4%)	9 (32.1%)	9 (32.1%)	7 (24.1%)	
Cystic kidney disease	4 (4.7%)	2 (7.1%)	0 (0%)	2 (6.9%)	
Others	15 (17.6%)	2 (7.1%)	8 (28.6%)	5 (17.2%)	0.284
Previous transplant	2 (2.4%)	1 (3.6%)	1 (3.6%)	0 (0.0%)	0.588
Pretransplant ESRD duration, years	3.0 (0.8–6.0)	2.5 (0.4–6.5)	3.0 (0.2–6.5)	3.0 (2.0–5.0)	0.669
Pretransplant therapy					0.204
Hemodialysis	61 (71.8%)	21 (75.0%)	20 (71.4%)	20 (69.0%)	
Peritoneal dialysis	14 (16.5%)	5 (17.9%)	2 (7.1%)	7 (24.1%)	
Preemptive transplantation	10 (11.8%)	2 (7.1%)	6 (21.4%)	2 (6.9%)	
**Transplant-related**					
Patients with preformed DSA	7 (8.3%)	2 (7.1%)	2 (7.1%)	3 (10.3%)	0.878
HLA mismatches					0.659
0	8 (9.4%)	3 (10.7%)	3 (10.7%)	2 (6.9%)	
1–2	1 (1.2%)	0 (0.0%)	1 (3.6%)	0 (0.0%)	
3–6	76 (89.4%)	25 (89.3%)	24 (85.7%)	27 (93.1%)	
ABO incompatible transplantation	6 (7.1%)	1 (3.6%)	3 (10.7%)	2 (6.9%)	0.58
Induction regimen					0.344
IL-2 receptor antagonist	82 (96.5%)	27 (96.4%)	26 (92.9%)	29 (100%)	
Antithymocyte globulin	3 (3.5%)	1 (3.6%)	2 (7.1%)	0 (0.0%)	
Initial immunosuppression					
Corticosteroids	85 (100%)	27 (100%)	12 (100%)	26 (100%)	NA
Tacrolimus	85 (100%)	27 (100%)	12 (100%)	26 (100%)	NA
Mycophenolate mofetil or mycophenolic acid	83 (97.6%)	27 (96.4%)	28 (100.0%)	28 (96.6%)	0.604
Azathioprine	2 (2.4%)	1 (3.6%)	0 (0.0%)	1 (3.4%)	0.604
Delayed graft function	10 (11.8%)	0 (0.0%)	2 (7.1%)	8 (27.6%)	0.004
Acute rejection at biopsy	12 (14.1%)	2 (7.1%)	4 (14.3%)	6 (20.7%)	0.371
Interval between transplant and sample collection, days	17.0 (15.0–19.0)	16.5 (15.0–18.5)	18.0 (16.5–19.0)	16.0 (15.0–19.0)	0.185
eGFR at baseline, mL/min per 1.73 m^2^	65.3 (53.5–83.8)	72.0 (60.7–85.8)	61.5 (53.9–85.6)	62.4 (44.7–75.1)	0.118

Values are given as mean ± standard deviation, median (interquartile range), or n (%). BMI, body mass index; DSA, donor-specific anti-HLA antibody; ESRD, end-stage renal disease; eGFR, estimated glomerular filtration rate. Tertile 1: 4.78–6.03 copies/mg Cr, Tertile 2: 6.03–6.64 copies/mg Cr, and Tertile 3: 6.64–8.22 copies/mg Cr.

### Urinary cell-free mtDNA level, renal function, and renal injury marker

**Figure 1:**
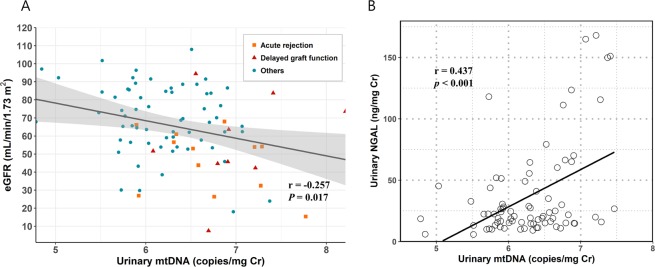
Association of urinary mtDNA with renal function and renal injury marker at baseline. (**A**) Correlation of urinary mtDNA level with eGFR. (**B**) Correlation of urinary mtDNA level with urinary NGAL.

First, we examined the associations of cell-free mtDNA level with eGFR and NGAL at baseline; NGAL has been extensively studied as a biomarker of post-transplant allograft function^19^. The urinary cell-free mtDNA level was negatively correlated with eGFR and positively correlated with urine NGAL level at the time of sample collection (Figs. 1A and 1B). In particular, patients with DGF or acute rejection showed relatively high levels of urinary mtDNA. The urinary nuclear DNA (nDNA) level also showed a comparable correlation with urine NGAL level, but the association with baseline eGFR was not significant (Fig. S1). These correlations were not observed with plasma cell-free nDNA and mtDNA levels (data not shown).

### Urinary cell-free mtDNA level and DGF

**Figure 2:**
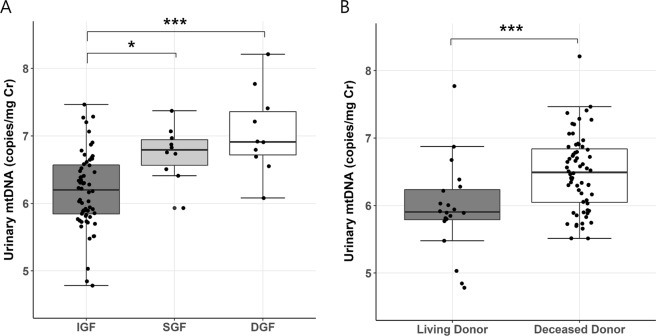
Urinary mtDNA level according to the early graft function and donor status. (**A**) IGF vs. SGF vs. DGF. (**B**) living donor transplantation vs. deceased donor transplantation. Data are presented as box-and-whisker plots; each box indicates the interquartile range. *P < 0.05, ***P < 0.001. IGF, immediate graft function; DGF, delayed graft function; SGF, slow graft function.

As shown in Table 1, more patients with DGF were included in higher urinary mtDNA tertile groups. Notably, there was a significant difference in urinary mtDNA level between subjects with DGF and IGF (P < 0.001, Fig. 2A). Furthermore, 10 patients with SGF were identified by the definition mentioned previously, and these patients showed higher urinary mtDNA levels than those with IGF. Urinary nDNA and NGAL were also elevated in subjects with DGF (Fig. S2). However, the differences between groups were more clearly in urinary mtDNA, which showed higher sensitivity in ROC curve analysis. These associations with DGF or SGF were not found in nDNA and mtDNA levels in plasma. Similarly, higher levels of urinary mtDNA were observed in deceased-donor kidney transplantation, a major known risk factor for DGF, compared to that in living-donor kidney transplantation (*P* < 0.001). These findings suggest that urinary mtDNA sensitively reflects the effects of IRI.

### Urinary cell-free mtDNA level and histological findings

Based on histological findings of the biopsy performed approximately 17 days after transplantation, patients were divided into 4 groups: no abnormalities, AR, acute tubular necrosis (ATN), and other injury. In our study, there were two cases of acute T cell-mediated rejection (Banff category 4) and 6 cases of acute antibody-mediated rejection (Banff category 2). Additionally, 4 cases revealed borderline changes (Banff category 3) in graft biopsy, but were treated with intravenous corticosteroid pulse therapy with strong clinical suspicion of rejection. These 12 patients were classified into the AR group. The other injury group included calcineurin inhibitor toxicity as well as nonspecific pathological findings, such as mesangial hypercellularity. The AR and ATN groups showed lower eGFR levels at baseline than those observed in the other groups (Table S1). The AR group more frequently had preformed donor-specific anti-HLA antibody (DSA), and the ATN group had more patients with DGF and higher BMI. The period between transplantation and biopsy was shorter in the AR group than in the other groups, but the difference was not statistically significant.

**Figure 3:**
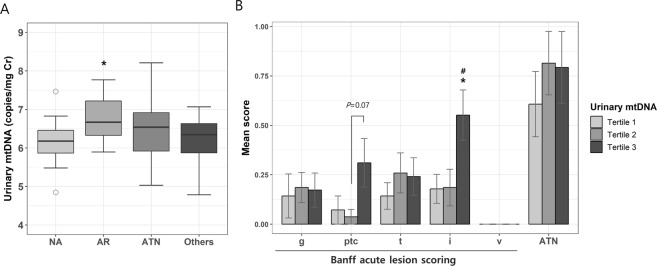
Relationship between urinary mtDNA and histological findings. (**A**) Urinary mtDNA level according to pathological diagnosis. *P < 0.05 vs. NA. (**B**) Banff acute lesion scoring according to the tertiles of urinary mtDNA. *P < 0.05 vs. tertile 1, ^#^*P* < 0.05 vs. tertile 2. Each box indicates the interquartile range in box-and-whisker plots. Error bars indicate the standard error of the mean. NA, no abnormalities; AR, acute rejection; ATN, acute tubular necrosis; Others, other injury.

When comparing results based on the histological diagnoses, a significant difference between groups was only observed for urinary mtDNA levels (Fig. 3A): urinary mtDNA levels were higher in the AR than those in the no abnormalities (*P* = 0.043) and in the other injury, although the latter was not statistically significant, as determined by post-hoc analysis. All other markers, including urinary nDNA, plasma nDNA and mtDNA, and urinary NGAL levels, showed no difference between the groups (Fig. S3). Next, we examined the acute lesion scoring based on Banff classification including interstitial inflammation (i), tubulitis (t), glomerulitis (g), and peritubular capillaritis (ptc) according to the urinary mtDNA level. Banff i and ptc scoring were shown to vary with the tertile of urinary mtDNA (Fig. 3B). Particularly, mean i score in tertile 3 was significantly higher than that in tertiles 1 or 2. On the other hand, g or t scores were not different between the tertile groups. All the abovementioned Banff components did not differ between plasma nDNA and mtDNA tertiles (data not shown).

### Urinary cell-free mtDNA level and short-term graft outcome

**Figure 4:**
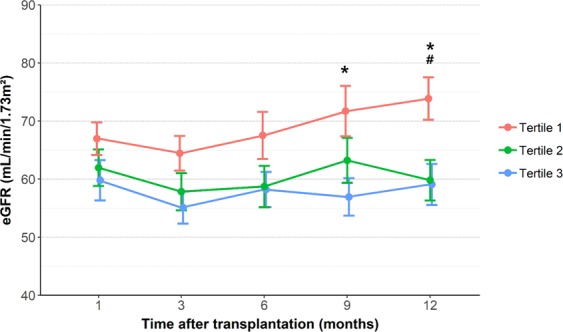
Evolution of kidney allograft function according to the tertiles of urinary mtDNA during 12 months after transplantation. *P < 0.05 vs. tertile 3, ^#^*P* < 0.05 vs. tertile 2. Error bars indicate the standard error of the mean.

The renal recovery time, defined in the Methods section, was positively correlated with urinary mtDNA levels (*P* < 0.001, Fig. S4). During the 1-year follow-up, three patients died due to serious infections, and none of the remaining subjects showed graft failure. As shown in Fig. 4, although there was no statistical difference in eGFR between urinary mtDNA tertile groups during the first 6 months, tertile 1 group showed significantly higher eGFR compared to the other tertile groups at 9 and 12 months post-transplant (*P* = 0.02 vs. tertile 3; *P* = 0.018 vs. tertile 2 and *P* = 0.012 vs. tertile 3, respectively); at 12 months, eGFR was 73.9 ± 18.5 mL/min/1.73 m^2^ in tertile 1, 59.8 ± 18.1 mL/min/1.73 m^2^ in tertile 2, and 59.1 ± 18.6 mL/min/1.73 m^2^ in tertile 3. Consistent with this finding, a mixed-effect regression model for eGFR adjusted for sex, age, time after transplantation, baseline graft function, and the presence of acute rejection or DGF showed significant interactions of urinary mtDNA tertiles with time (Table 2). In other words, our final model indicated that lower tertiles of urinary mtDNA had better graft function with time.

**Table 2:** Results of final mixed-effect regression model for allograft function during follow-up period.

Variable	Estimate	95% CI	p-value
Intercept	53.22	36.07 to 70.35	<0.001
Donor age (yr)	−0.24	−0.43 to −0.06	0.017
Donor sex	1.47	−2.58 to 5.51	0.502
Recipient age (yr)	−0.09	−0.29 to 0.11	0.426
Recipient sex	0.64	−3.80 to 5.08	0.793
Deceased donor	−0.74	−6.06 to 4.58	0.800
eGFR at baseline	0.47	0.37 to 0.56	<0.001
Interval between transplant and sample collection (days)	−0.58	−1.18 to 0.06	0.082
Acute rejection at biopsy	3.29	−3.12 to 9.69	0.352
Delayed graft function	3.33	−3.25 to 9.91	0.358
Tertile2	−2.20	−8.11 to 3.75	0.494
Tertile3	2.65	−3.39 to 8.76	0.421
Time (months)	1.98	0.59 to 3.37	<0.001
Time * Tertile2 (interaction)	−1.74	−3.71 to 0.24	0.091
Time * Tertile3 (interaction)	−2.42	−4.35 to −0.48	0.017

eGFR, estimated glomerular filtration rate; values are given as mean ± standard deviation or n (%).

## Discussion

In this study, we investigated the clinical implications of cell-free mtDNA quantified with qPCR from urine and plasma samples of 85 kidney transplant recipients during the early post-transplant period. We analyzed the association of cell-free mtDNA with multiple clinical parameters and histological findings in simultaneously performed graft biopsy. Of the tested markers, the urinary mtDNA level was found to be highly sensitive to renal graft injury; in particular, urinary mtDNA increased significantly in subjects with DGF. Also, acute rejection was associated with higher levels of urinary mtDNA. Although at baseline urinary mtDNA level was weakly correlated with graft function and no significant difference between tertile groups was observed, the lowest tertile group showed better renal function at 12 months post-transplant compared with the higher tertile groups.

Renal IRI is a major cause of AKI and post-transplant allograft dysfunction^20,21^, which can clinically manifest as DGF. Ischemia-reperfusion is known to induce ROS generation, microvascular injury, inflammation, and cell death, resulting in inferior graft outcome^6,7^. In these pathophysiologic aspects of IRI, the innate immune system is involved through numerous mechanisms and DAMPs play a pivotal role in the activation of innate immunity. Mitochondria are also a potent source of DAMPs, and mitochondrial damage is closely associated with IRI^22^. Mitochondrial damage can contribute to AKI progression through multiple pathophysiological processes, including opening of the mitochondrial permeability transition pore, ROS release, ATP depletion, and mitochondrial DAMPs including N-formyl peptides, cytochrome c, and mtDNA^23^. Among these processes, cell-free mtDNA is actively under investigation as a potential predictor and causal factor of kindey injury^14–16^. Although recent studies showed that urinary mtDNA could be associated with post-transplant allograft function^17,18^, clinical implications of urinary mtDNA to date have been mainly evaluated in AKI rather than in kidney transplantation. Whitaker *et al*. showed that urinary mtDNA levels correlated well with ischemic time and AKI progression after cardiac surgery^15^. Similarly, Hu *et al*. showed the significant association between urinary mtDNA and the severity of AKI in patients with sepsis^24^ and surgical critical illness^16^. In these studies on AKI, urinary mtDNA level showed significant inverse correlations with renal tissue mtDNA level, ATP contents, and expression of mitochondrial genes such as PGC-1α, NDUFB8^15,24^, suggesting mitochondrial dysfunction in AKI. Decreased mitochondrial gene expression and ATP depletion are also related with altered mitochondrial homeostasis and impaired renal repair processes after AKI^23^.

In the present study, cell-free mtDNA was measured approximately 2 weeks after transplantation, a period in which grafts mildly affected by IRI could achieve stable function. Although plasma mtDNA rapidly decreases to baseline with a short half-life^25^, dysfunctional mitochondria in renal tubular epithelial cells can continue to release mtDNA fragments into urine^26^. Therefore, a high level of urinary mtDNA beyond 1 week after transplantation may suggest that urinary mtDNA may be associated with persistent mitochondrial dysfunction rather than immediate release by IRI^24^. Impaired antioxidant defense system of mitochondria increases ROS generation, which causes mtDNA damage^27^. As a result, mtDNA fragments are released from mitochondria via mitochondrial permeability transition pore or necroptosis^28,29^. Persistent mitochondrial damage after AKI has been reported in several literatures^30,31^. Szeto *et al*., demonstrated that mitochondrial damage in podocytes and proximal tubular cells persisted 9 months after ischemic insult, contributing to inflammation and chronic renal injury^31^. Because mitochondrial bioenergetic function is crucial for renal repair and recovery, persistent mitochondrial dysfunction leads to renal tubular cell epithelial-mesenchymal transition and renal fibrosis^32^. Furthermore, urinary mtDNA can be increased even in patients with chronic kidney disease^26,33^ Chronic inflammation caused by innate immune activation also plays a crucial role in renal fibrosis after acute ischemia^34,35^. Mitochondrial DNA-mediated inflammation is known to be driven by the activation of TLR9, NOD-like receptor pyrin domain-containing-3 (NLRP3) inflammasome, and the simulator of interferon genes signaling^36^. MtDNA can directly activate the NLRP3^37^, and the inhibition of mtDNA release suppresses inflammasome formation^38^, suggesting a positive feedback between mtDNA and NLRP3 inflammasome. Tsuji *et al*. demonstrated that mtDNA stimulated cytokine production and renal mitochondrial injury via the TLR9 pathway^39^. Likewise, the association between urinary mtDNA and allograft functions at 9- and 12-month post-transplantation could be explained in part by these mechanisms, although the elucidation of detailed mechanistic pathway requires further investigation. Nevertheless, our results provide insights into the role of urinary cell-free mtDNA in renal allograft outcome.

There are several other considerations about the findings of urinary mtDNA during early post-transplant period. First, deceased donor transplantation, DGF, and even SGF are significantly associated with increased urinary mtDNA release, but not with plasma mtDNA, and more strongly than urinary nDNA. It has been shown that, compared with nDNA, mtDNA is more vulnerable to oxidative stress caused by ROS in IRI due to a lack of repair mechanism and the absence of histone protection^40^. Accordingly, our findings, along with those of a recent study of urinary mtDNA on DGF^18^, indicate that urinary mtDNA could be a sensitive indicator for IRI. Second, immunologic injury such as acute rejection also increased the urinary mtDNA level, suggesting that ongoing injury other than IRI can elevate the urinary mtDNA level. Specifically, tubulointerstitial inflammation involved in the process of acute rejection might contribute to the mitochondrial damage. The proximal tubule, which is a major target in ischemic AKI, are very rich in mitochondria required for the generation of ATP^23,27^, which supports these findings and our correlations between urinary mtDNA levels and NGAL, a known renal tubular injury marker. However, despite the association with DGF, the urinary mtDNA level in ATN group was not elevated and ATN grades according to the tertiles were not different. Because most of the enrolled subjects underwent early protocol biopsy rather than indicated biopsy, the ATN group included many subclinical patients with good graft function; of all 26 ATN subjects, the majority (62%) had mild ATN. Furthermore, some of the patients may have recovered histologically from post-transplant ATN at the time of biopsy.

Our results indicated relatively weak correlations between urinary mtDNA and eGFR at baseline. However, mtDNA levels were well correlated with post-transplant renal recovery time. Further, when examined over time, allograft functions at 9 and 12 months post-transplant were significantly different between the tertile groups of mtDNA independent of the presence of DGF or acute rejection, showing significantly better graft outcome in the lowest tertile group. These results revealed the association of mtDNA with short or intermediate-term graft outcome and indicated that mtDNA levels may provide prognostic information. Further, mtDNA can serve as a surrogate markers of mitochondria dysfunction and offer the possibility of its application as a therapeutic target for renal allograft injury. Given that few studies have addressed the impact of mtDNA on graft prognosis beyond the immediate post-transplant period, our study can contribute towards understanding the clinical relevance of cell-free mtDNA.

Our study has several limitations. The total number of subjects was small. In particular, a small number of patients with acute rejection was included and analyzed together with borderline changes. It was difficult to evaluate robust graft outcomes such as graft failure due to the small sample size and a short follow-up period. Moreover, our study lacks data on changes in urinary mtDNA levels during the follow-up period. To evaluate the differences in prognosis according to cell-free mtDNA levels, serial measurements would be helpful. Nevertheless, our results clearly showed that urinary cell-free mtDNA level is associated with graft injury early after transplantation and seems to be predictive of subsequent graft function, although further confirmatory studies are warranted.

In conclusion, urinary cell-free mtDNA levels during the early post-transplant period are significantly associated with the presence of DGF, acute rejection in graft biopsy, and short-term post-transplant graft function.

## Methods

### Study population and design

We enrolled a total of 85 renal transplant recipients who underwent kidney transplantation and had been followed up for >3 months at Kyung Hee University Hospital at Gangdong from January 2012 to February 2015. All except one patient had graft biopsy performed approximately 2–3 weeks after transplantation. Blood (n = 78) and urine (n = 85) samples were collected early in the morning of graft biopsy. Patients with missing urine samples or active infection were excluded. Foley catheters were removed on postoperative day 5 according to the center’s protocol. All enrolled subjects were categorized on the basis of urinary mtDNA levels into tertile 1 (4.78–6.03 copies/mg Cr), tertile 2 (6.03–6.64 copies/mg Cr), and tertile 3 (6.64–8.22 copies/mg Cr). Post-transplant renal function according to the tertiles was evaluated during the follow-up period of 12 months. We also examined the association of urinary mtDNA levels with clinical information and pathological findings^41^. Follow-up information on graft status was collected every 3 months for the first year after transplantation. The recipients’ data including age, sex, laboratory findings, and donor information were collected from electronic medical records. The Chronic Kidney Disease Epidemiology Collaboration (CKD-EPI) equation was used to calculate estimated glomerular filtration rate (eGFR) of the patients^42^. DGF was defined as needing dialysis within 7 days of transplantation. Slow graft function (SGF) and immediate graft function (IGF) were defined as reductions in serum creatinine of <20% or >20% within the first 24 h post-transplant, respectively^43^. Additionally, we defined renal recovery time as the period from the time of transplantation until serum creatinine levels reached their nadir and were stable for more than 7 days. All studied transplant recipients and organ donors provided written informed consent prior to participation in the study. No organs or tissues were procured from prisoners, and all of them was obtained from patients who received kidney transplantation at Kyung Hee University Hospital at Gangdong. The study was conducted in accordance with the Declaration of Helsinki, and the sample collection and application for research purpose were approved by the local institutional review board (#2008-01-035 and #2012-01-030, Institutional Review Board of Kyung Hee University Hospital) and were registered with the Clinical Research Information Service (KCT0001010).

### Cell-free DNA extraction and quantification

Approximately 10 mL of blood samples drawn in heparinized tubes and 50 ml of urine samples were collected from the participants. Each blood and urine sample was centrifuged at 850 *g* for 30 min and at 2000 *g* for 20 min, respectively, and plasma and urine supernatant samples were isolated and stored at -80 °C until cell-free DNA (cfDNA) measurement. Cell-free DNA was extracted from 200 µL of plasma and 400 µL of urine supernatant using a QIAamp DNeasy Blood and Tissue kit (Qiagen, Valencia, CA, USA). Cell-free nuclear and mitochondrial DNA concentrations were measured by quantitative real-time polymerase chain reaction (RT-PCR) targeting the human lipoprotein lipase (LPL) gene and human NADH1 dehydrogenase subunit 1 (ND1) gene, respectively, using a StepOnePlus real-time PCR system (Applied Biosystems, Foster City, MA, USA). The sequences of the ND1 primers were as follows: forward 5′-ATACCCATGGCCAACCTCCT-3′, reverse 5′-GGGCCTTTGCGTAGTTGTAT-3′; and the sequences of the LPL primers were as follows: forward 5′-CGAGTCGTCTTTCTCCTGATGAT-3′, reverse 5′-TTCTGGATTCCAATGCTTCGA-3′. Standard DNA fragments for ND1 and LPL were synthesized by Integrated DNA Technologies (IDT, Coralville, IA, USA) for absolute quantification. Urinary neutrophil gelatinase-associated lipocalin (NGAL) was measured using a Human Lipocalin-2/NGAL Quantikine ELISA kit (R&D Systems, Minneapolis, MN, USA), according to the manufacturer’s protocol. Urine creatinine concentration was measured using the Creatinine Parameter Assay kit (R&D Systems) and urinary cell-free nDNA and mtDNA copy numbers and NGAL levels were corrected for measured urine creatinine.

### Pathological description

All biopsy specimens were examined by experienced renal pathologists blinded to patients’ clinical information. Renal allograft pathological features were described and graded as per the Banff 2013 classification^41^. Based on the pathologists’ reports, histological diagnosis was classified into four categories (no abnormalities, AR, ATN, and other injury) to determine the association with the cell-free mtDNA level. ATN was diagnosed based on histological findings such as epithelial swelling with lucent cytoplasm, loss of brush border, and epithelial flattening^44^ and scored according to the extent of lesion (0: absence of lesion, grade 1: <25% lesions, grade 2: 25–50% lesion, and grade 3: ≥50% lesions)^45^.

### Statistical analysis

All statistical analyses were performed using the R software (version 3.5.1; R Foundation for Statistical Computing). DNA copy numbers were logarithmically transformed before analysis. Comparisons of baseline characteristics across tertiles were made using ANOVA or Kruskal–Wallis test for continuous variables and χ^2^ test or Fisher-exact for categorical variables, depending on the normality of the distribution. The correlation of cell-free nDNA and mtDNA levels with eGFR or urinary NGAL was assessed using Spearman correlation test. The comparisons according to the early graft function and donor status were carried out using the Mann–Whitney U test and Kruskal–Wallis test. Linear mixed effects modeling was also performed to examine the longitudinal change of graft function with lme4 package (version 1.1–20) in R. Differences with P-values less than 0.05 were considered statistically significant.

## Supplementary information

Supplementary Figures and table
