# Supplementary material for: Clinical relevance of cell-free mitochondrial DNA during the early postoperative period in kidney transplant recipients
Source: Sci Rep. 2019 Dec 9;9:18607. doi: 10.1038/s41598-019-54694-x (PMC6901568; doi:10.1038/s41598-019-54694-x)
Supplement: Supplementary file 1 — Supplementary Figures and table [file 41598_2019_54694_MOESM1_ESM.docx]

Supplementary Information for

**Clinical relevance of cell-free mitochondrial DNA during the early postoperative period in kidney transplant recipients**

Kipyo Kim, Haena Moon, Yu Ho Lee, Jung-Woo Seo, Yang Gyun Kim, Ju-Young Moon, Jin Sug Kim, Kyung-Hwan Jeong, Tae Won Lee, Chun-Gyoo Ihm, and Sang-Ho Lee

Corresponding author: Sang Ho Lee, MD. PhD

Email: lshkidney@khu.ac.kr

This file includes:

Figure S1 to S4

Tables S1

**Figure S1.** Association of urinary nDNA with renal function and renal injury marker at baseline. A, correlation of urinary nDNA with eGFR. B, correlation of urinary nDNA with urinary NGAL.
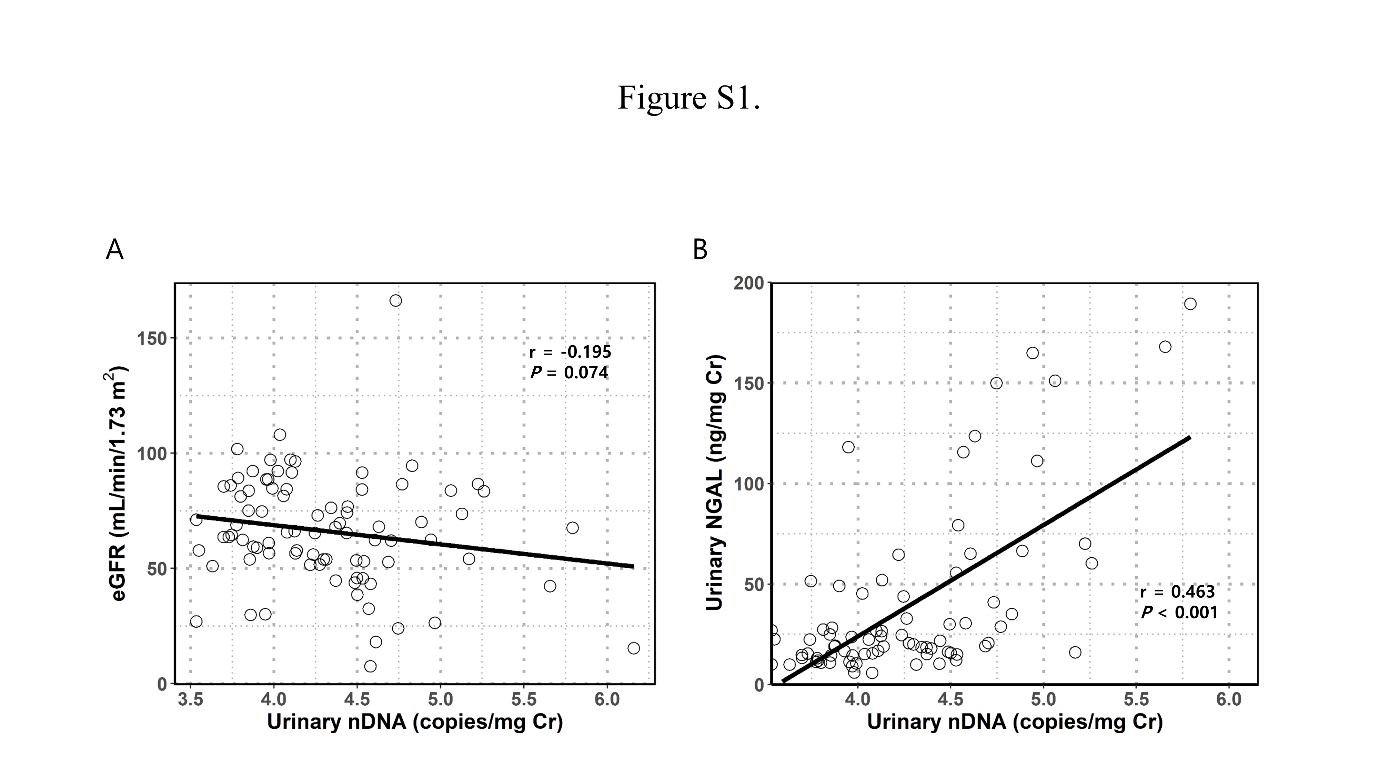


**Figure S2.** Association of urinary nDNA and NGAL with early graft function. A, receiver-operating characteristic curves of urinary nDNA, mtDNA, and NGAL for DGF. B, C, urinary nDNA and NGAL according to the early graft function. D, E, plasma nDNA and mtDNA according to the early graft function. (**P<0.01, *P<0.05)
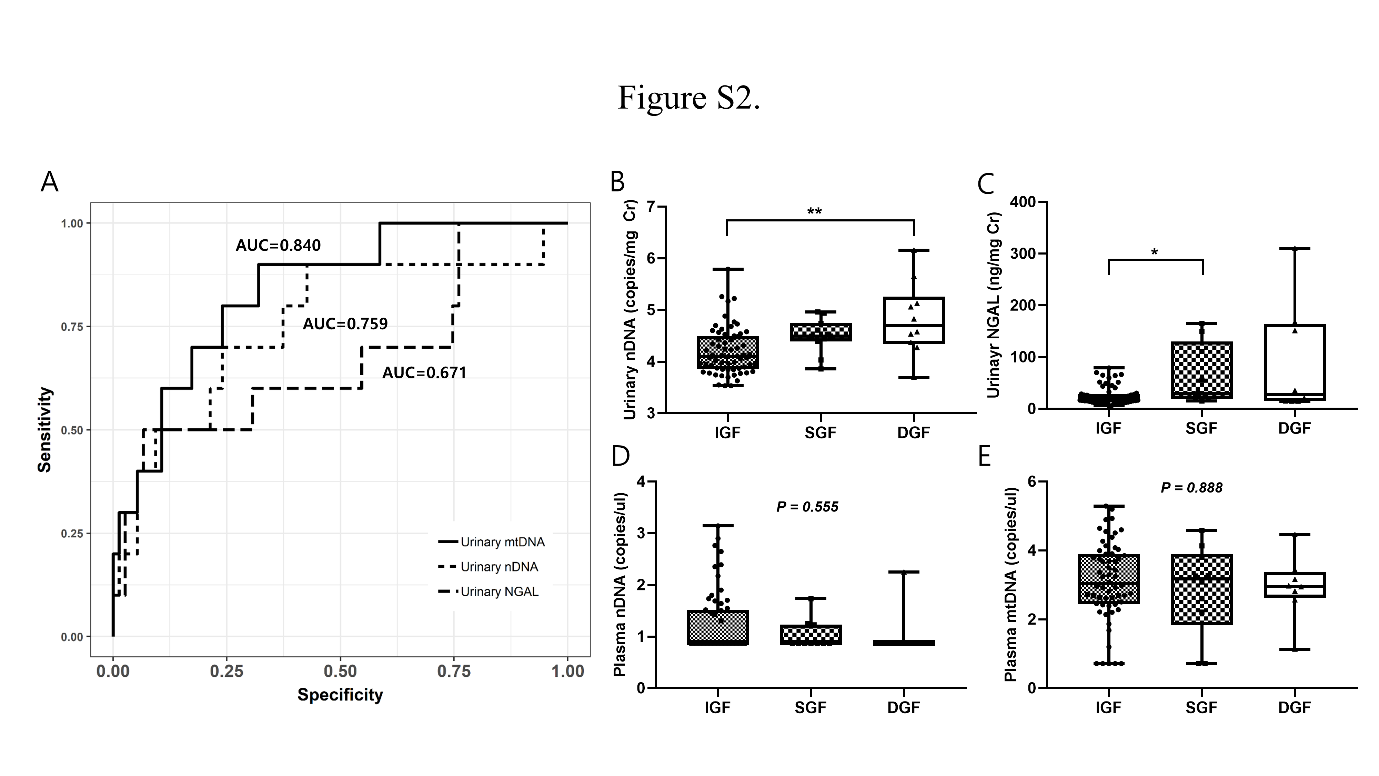


Figure S3. Relationship between pathological diagnosis and urinary nDNA. (A), urinary NGAL (B), plasma nDNA (C), and plasma mtDNA (D). Each box indicates the interquartile range in box-and-whisker plots. NA, no abnormalities; AR, acute rejection; ATN, acute tubular necrosis; Others, other injury.


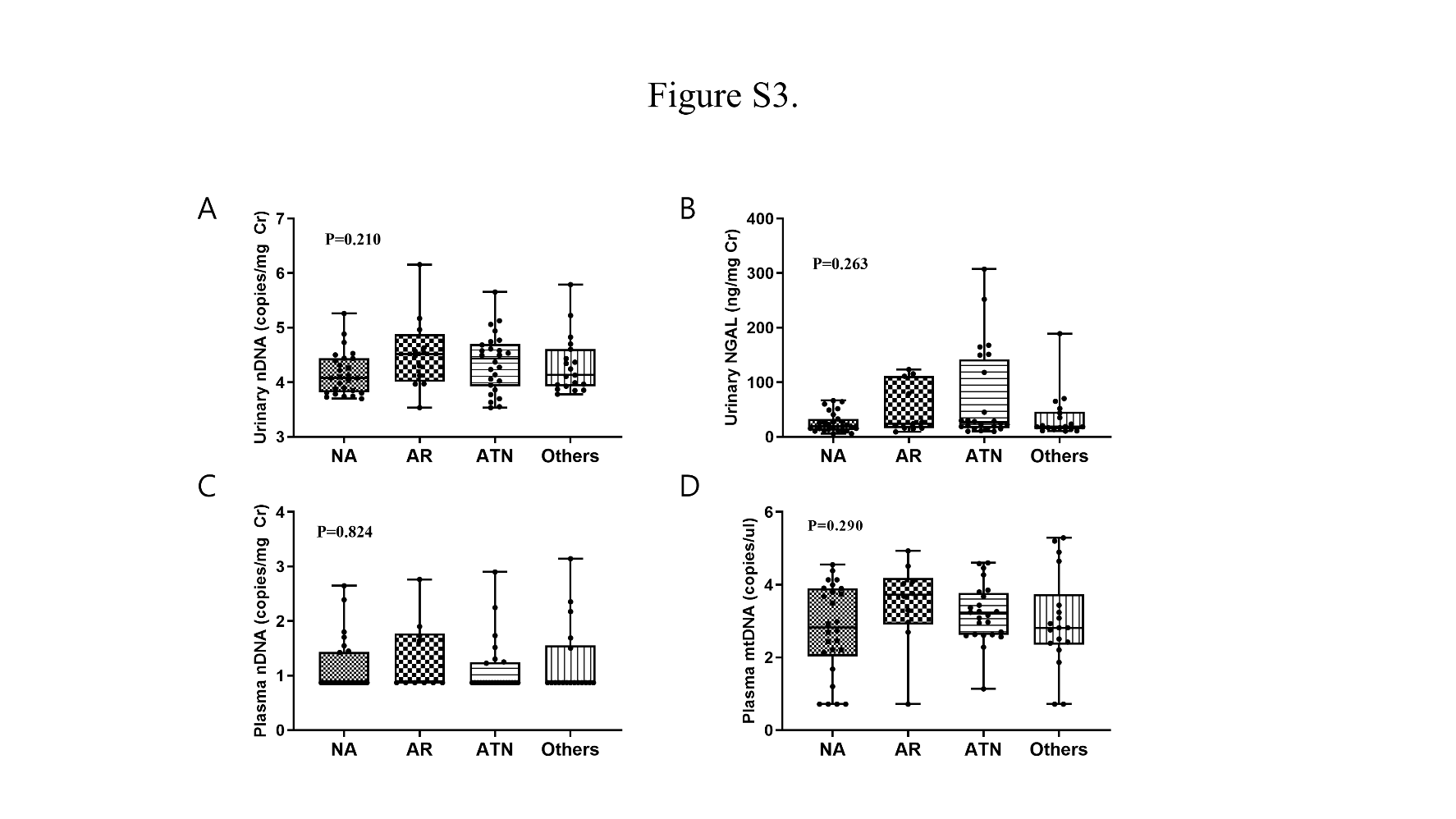


Figure S4. Correlation of urinary mtDNA levels with renal recovery time.


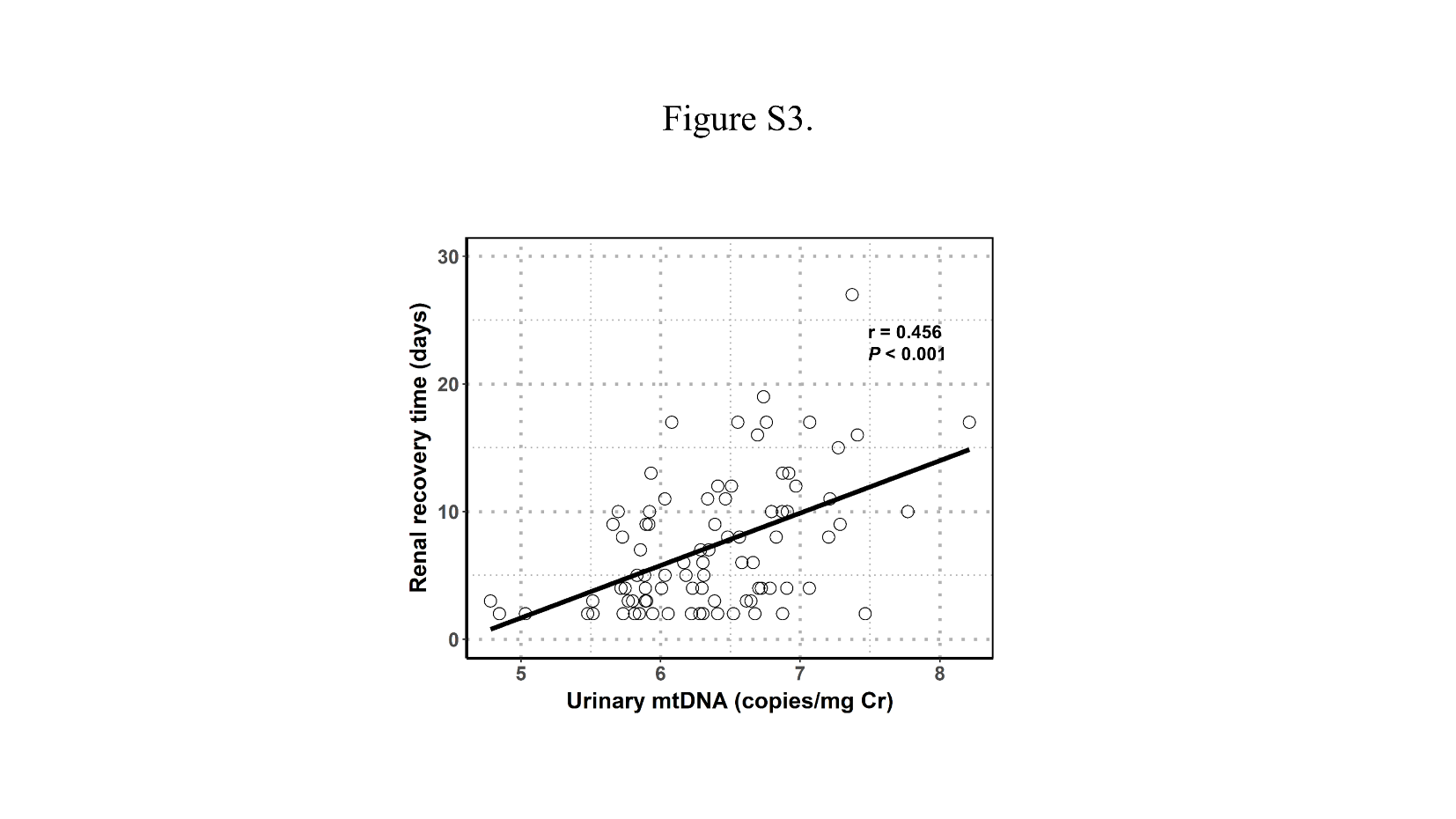


**Table S1.** Clinical characteristic of study population according to the pathologic diagnosis.

|  | Total  (n=84) | No abnormalities (n=27) | Acute rejection  (n=12) | ATN (n=26) | Other injury  (n=19) | *P* |
| --- | --- | --- | --- | --- | --- | --- |
| **Donor** |  |  |  |  |  |  |
| Age, years | 47.3 ± 10.8 | 45.4 ± 11.9 | 48.9 ± 11.3 | 48.0 ± 9.9 | 47.9 ± 10.4 | 0.730 |
| Male sex | 48 (57.1%) | 17 (63.0%) | 5 (41.7%) | 15 (57.7%) | 11 (57.9%) | 0.670 |
| Donor type |  |  |  |  |  | 0.558 |
| Living | 20 (23.8%) | 9 (33.3%) | 2 (16.7%) | 5 (19.2%) | 4 (21.1%) |  |
| Deceased | 64 (76.2%) | 18 (66.7%) | 10 (83.3%) | 21 (80.8%) | 15 (78.9%) |  |
| **Recipient** |  |  |  |  |  |  |
| Age, years | 50.0 (41.0–56.0) | 49.0 (37.0–52.5) | 56.0 (49.0–60.5) | 51.0 (43.0–57.0) | 49.0 (40.5–55.5) | 0.082 |
| Male sex | 59 (70.2%) | 19 (70.4%) | 6 (50.0%) | 22 (84.6%) | 12 (63.2%) | 0.146 |
| BMI, kg/m^2^ | 21.9 (20.1–24.3) | 21.9 (20.5–23.6) | 22.2 (19.5–23.9) | 24.1 (21.3–27.1) | 20.7 (19.2–22.0) | 0.02 |
| Cause of ESRD |  |  |  |  |  | 0.252 |
| Diabetes | 18 (21.4%) | 6 (22.2%) | 3 (25.0%) | 7 (26.9%) | 2 (10.5%) |  |
| Hypertension | 23 (27.4%) | 7 (25.9%) | 2 (16.7%) | 10 (38.5%) | 4 (21.1%) |  |
| Glomerulonephritis | 25 (29.8%) | 9 (33.3%) | 2 (16.7%) | 5 (19.2%) | 9 (47.4%) |  |
| Cystic kidney disease | 4 (4.8%) | 0 (0%) | 2 (16.7%) | 2 (7.7%) | 0 (0%) |  |
| Others | 14 (16.7%) | 5 (18.5%) | 3 (25.0%) | 2 (7.7%) | 4 (21.1%) |  |
| Previous transplant | 2 (2.4%) | 1 (3.7%) | 0 (0.0%) | 0 (0.0%) | 1 (5.3%) | 0.613 |
| Pretransplant ESRD duration, years | 3.0 (0.8–6.0) | 3.0 (0.2–4.5) | 3.0 (0.8–6.5) | 3.5 (1.0–6.0) | 4.0 (1.5–7.0) | 0.443 |
| Pretransplant therapy |  |  |  |  |  | 0.796 |
| Hemodialysis | 60 (71.4%) | 17 (63.0%) | 9 (75.0%) | 21 (80.8%) | 13 (68.4%) |  |
| Peritoneal dialysis | 14 (16.7%) | 6 (22.2%) | 1 (8.3%) | 3 (11.5%) | 4 (21.1%) |  |
| Preemptive transplantation | 10 (11.9%) | 4 (14.8%) | 2 (16.7%) | 2 (7.7%) | 2 (10.5%) |  |
| **Transplant-related** |  |  |  |  |  |  |
| Patients with preformed DSA | 7 (8.3%) | 1 (3.7%) | 4 (33.3%) | 1 (3.8%) | 1 (5.3%) | 0.009 |
| HLA mismatches |  |  |  |  |  | 0.716 |
| 0 | 7 (8.3%) | 2 (7.4%) | 0 (0.0%) | 3 (11.5%) | 2 (10.5%) |  |
| 1-2 | 1 (1.2%) | 1 (3.7%) | 0 (0.0%) | 0 (0.0%) | 0 (0.0%) |  |
| 3-6 | 76 (90.5%) | 24 (88.9%) | 12 (100.0%) | 23 (88.5%) | 17 (89.5%) |  |
| ABO incompatible transplantation | 6 (7.1%) | 3 (11.1%) | 0 (0.0%) | 0 (0.0%) | 3 (15.8%) | 0.127 |
| Induction regimen |  |  |  |  |  | 0.682 |
| Antithymocyte globulin | 3 (3.6%) | 1 (3.7%) | 1 (8.3%) | 1 (3.8%) | 0 (0.0%) |  |
| Basiliximab | 81 (96.4%) | 26 (96.3%) | 11 (91.7%) | 25 (96.2%) | 19 (100.0%) |  |
| Initial immunosuppression |  |  |  |  |  |  |
| Corticosteroids | 85 (100%) | 27 (100%) | 12 (100%) | 26 (100%) | 19 (100%) | NA |
| Tacrolimus | 85 (100%) | 27 (100%) | 12 (100%) | 26 (100%) | 19 (100%) | NA |
| Mycophenolate mofetil or Mycophenolic acid | 82 (97.6%) | 25 (92.6%) | 10 (83.3%) | 26 (100.0%) | 16 (84.2%) | 0.284 |
| Azathioprine | 2 (2.4%) | 2 (7.4%) | 1 (8.3%) | 0 (0.0%) | 2 (10.5%) | 0.284 |
| Delayed graft function | 10 (11.9%) | 0 (0.0%) | 1 (8.3%) | 8 (30.8%) | 1 (5.3%) | 0.004 |
| Interval between transplant and biopsy, days | 17.5 (15.0–19.0) | 17.0 (16.0–17.5) | 15.5 (10.5–17.5) | 18.0 (16.0–18.5) | 18.0 (16.0–18.5) | 0.149 |
| eGFR at baseline, mL/min per 1.73 m^2^ | 65.3 (53.2–83.7) | 75.1 (64.1–87.6) | 53.5 (29.7–58.8) | 53.1 (43.2–68.7) | 76.2 (65.3–88.6) | <0.001 |

Values are given as mean ± standard deviation or n (%). BMI, body mass index; DSA, donor-specific anti-HLA antibody; ESRD, end-stage renal disease; eGFR, estimated glomerular filtration rate. § P < 0.001 vs. normal findings and other injury; ¶ P < 0.01 vs normal findings and P < 0.001 vs other injury.
